# Commentary: Effect of curcumin nanoparticles on proliferation and migration of mouse airway smooth muscle cells and airway inflammatory infiltration

**DOI:** 10.3389/fphar.2024.1432397

**Published:** 2024-07-24

**Authors:** Fabien Beaufils, Patrick Berger

**Affiliations:** ^1^ Centre de Recherche Cardio-thoracique de Bordeaux, INSERM U1045, Bordeaux Imaging Center, University Bordeaux, Pessac, France; ^2^ CHU Bordeaux, Département de Pédiatrie, CIC-P 1401, Service d’Anatomopathologie, Service d’Exploration Fonctionnelle Respiratoire, Bordeaux, France; ^3^ INSERM, Centre de Recherche Cardio-thoracique de Bordeaux, U1045, Centre d’Investigation Clinique (CIC-P 1401), Pessac, France

**Keywords:** asthma, bronchial remodeling, smooth muscle, proliferation, migration

## Introduction

We read with interest the recent publication by Ma et al., Effect of curcumin nanoparticles on proliferation and migration of mouse airway smooth muscle cells and airway inflammatory infiltration (Ma et al., 2024) highlighting curcumin nanoparticles (CUR-NPs) as a potential new treatment of bronchial remodeling in asthma. Bronchial remodeling is a hallmark of asthma associated with both worse clinical outcomes and airflow obstruction related to structural alterations and thickening of the bronchial wall (Varricchi et al., 2022). Among the structural alterations of the bronchial wall, the increase in airway smooth muscle (ASM) mass appears as a key player by contributing to irreversible airflow limitation, poor symptom control and lack of response to treatment (Girodet et al., 2016; Varricchi et al., 2022). In asthma, ASM remodeling has been related to an increased ASM cell (ASMC) proliferation and migration enhanced by several factors (e.g., transforming growth factor (TGF)-β1) (Bara et al., 2010). In addition, ASMCs can produce themselves TGF-β1, collagen and fibronectin, also involved in bronchial fibrosis (Bara et al., 2010; Camoretti-Mercado and Lockey, 2021; Varricchi et al., 2022). However, usual anti-asthmatic treatments (*i.e.*, corticosteroids and β2-adrenergic receptor agonists) remain unable to decrease ASMC proliferation and migration *in vitro* (Roth et al., 2004; Trian et al., 2007; Bara et al., 2010) as well as ASM mass *in vivo* in asthma (Camoretti-Mercado and Lockey, 2021), reinforcing the need of studies such as that of Ma et al. on curcumin, to identify new treatment that specifically target the ASM remodeling.

## A novel method of curcumin administration

Curcumin is a polyphenolic compound derived from the curcuma plant exhibits anti-inflammatory and antioxidant properties, whose use in various forms (e.g., curcumin-containing poly (lactic-co-glycolic acid)-based microscale discoidal polymeric particles, curcumin-loaded niosomes or liposomal curcumin) has demonstrated promising preclinical results in asthma (Lelli et al., 2017; Ng et al., 2018; Park et al., 2020; Wong et al., 2020; Panknin et al., 2023). However, the poor bioavailability of curcumin delivered by oral ingestion may have limited its efficacy to improve lung function or symptoms in randomized clinical trials (Kim et al., 2011; Lelli et al., 2017; Panknin et al., 2023). Thus, the used of a novel method of administration, curcumin-coupled nanoparticles (CUR-NPs), represented a major advance and the strength of the study of Ma et al. Indeed, the authors clearly demonstrated that coupling curcumin to nanoparticles improved both curcumin intracellular uptake and accumulation *in vitro* (Ma et al., 2024). What is missing however, is the comparison of curcumin bioavailability in the lungs of mice treated either by coupled or uncoupled curcumin to fully convinced that CUR-NPs could fix the poor bioavailability issues identified in clinical trials (Kim et al., 2011; Lelli et al., 2017; Panknin et al., 2023).

## Effects of curcumin-coupled nanoparticles

The other strong value of the study of Ma et al., is the *in vivo* validation of *in vitro* results. Indeed they also demonstrated, in lungs tissue from sensitized mice *in vivo,* that CUR-NPs decreased the protein expression of TGF-β1, Signal transducer and activator of transcription 3 (STAT3) and Connective tissue growth factor (CTGF) (Ma et al., 2024), whose roles in airway remodeling have been demonstrated (Bara et al., 2010; Gao et al., 2014; Gavino et al., 2016). They also showed, *in vitro*, that CUR-NPs may have a potential effect on both proliferation and migration of mouse-isolated tracheal cells described as ASMC. These effects of CUR-NPs are particularly relevant since both ASMC proliferation and migration are key mechanisms involved in ASM remodeling in asthma (Bara et al., 2010; Camoretti-Mercado and Lockey, 2021). Indeed, ASMC from asthmatic patients proliferate faster than those from controls (Bara et al., 2010; Esteves et al., 2021). This increased proliferation is mitochondria-dependent (Bara et al., 2010; Esteves et al., 2021) and can be enhanced by several factors increased in asthma such as growth factors/cytokines (e.g., TGFβ-1), inflammatory mediators (e.g., cysteinyl leukotriene) or enzymes (e.g., tryptase) (Bara et al., 2010; Trian et al., 2015; Camoretti-Mercado and Lockey, 2021). In addition, most of these mediators (e.g., TGFβ-1) can enhance the migration of ASMCs increasing ASM remodeling (Bara et al., 2010) and promote others characteristics of bronchial remodeling such as bronchial fibrosis (Camoretti-Mercado and Lockey, 2021; Varricchi et al., 2022). However, potential limitations of the study need to be considered.

## Characterization of cells

Firstly, the authors described in the methods section the extraction and culture of murine ASMCs from cervical trachea and lung tissues (Ma et al., 2024) but there is no description regarding cell phenotype assessment to confirm the smooth muscle phenotype, as usually performed (Trian et al., 2007; Beaufils et al., 2021; Celle et al., 2022). This lack of information raises the question of what cell type has been studied, and how the authors concluded that these were indeed smooth muscle cells. In our hand, culturing murine ASMCs is more difficult than human ASMCs, because murine fibroblasts are more prone to proliferate than ASMCs.

## Effect of CUR-NPs on isolated cell proliferation and migration

Secondly, ASMC proliferation was assessed using the Cell Counting Kit (CCK)-8 to the number of living cells. In the present study, this methods demonstrated approximately a doubling of the number of living cells after 48 h in cells stimulated by TGFβ-1 for 48 h compared to unstimulated cells or stimulated cells treated with CUR-NPs at 40 or 50 µM (Ma et al., 2024). Thus, this method confirmed an increased cell proliferation induced by TGFβ-1. However, the difference between TGFβ-1/CUR-NPs-treated cells and TGFβ-1-treated cells can suggest either a decreased cell proliferation, as mentioned by the authors, or an increased cell death, hence a possible toxicity, induced by CUR-NPs. Without any data regarding the effect of CUR-NPs on unstimulated cells to assess their toxicity, and/or specific assessment of cell death, it is thus difficult to conclude that CUR-NPs decreased the TGFβ-1 induced ASMC proliferation. In addition, if ASMC proliferation was effectively reduced or cell death increased upon CUR-NPs (40 µM) after 48 h of culture, the increased recovery of the gap by unstimulated ASMCs during migration assays using wound healing and transwell assays may be difficult to interpret. Indeed, after 48 h, the increased recovery of the gap by TGFβ-1-stimulated cells may result from an increased number of cells due to an increased proliferation or a decreased cell death of these cells compared with CUR-NPs-treated ASMCs instead of a difference in cell migration. As consequence, an actual decrease in ASMC migration remains to be demonstrated by repeating the migration assays over a shorter time (*e.g.*, 24 h), less than the doubling time of both CUR-NPs-treated and untreated cells, and after comparison of cell death between CUR-NPs-treated and untreated cells.

## Effect of CUR-NPs on airway remodeling

Thirdly, in their conclusion, the authors stated that CUR-NPs can effectively reverse airway remodeling in asthma (Ma et al., 2024). However, in their study, Ma et al. (2024) did not quantify the ASM mass to compare its remodeling between controls and asthmatic mice or between untreated and CUR-NPs-treated asthmatic mice. They also did not quantify the airway fibrosis whereas they clearly demonstrated the impacted CUR-NPs on TGFβ-1, a major player in this key aspect of bronchial remodeling. This lack of data regarding the ASM mass and airway fibrosis in mice limits the validity of their conclusion that CUR-NPs to effectively target airway remodeling *in vivo* despite the others promising *in vitro* and *in vivo* results. Indeed, among the several pharmacological components able to impact ASMC proliferation, migration and/or cell death *in vitro,* only two (*i.e*., gallopamil and fevipiprant) appeared able to decrease ASM mass *in vivo* (Camoretti-Mercado, 2009; Bara et al., 2010; Girodet et al., 2015; Saunders et al., 2019). In addition, bronchial thermoplasty has also been shown to decrease ASM mass *in vivo* (Cox et al., 2007; Girodet et al., 2015; Saunders et al., 2019).

In conclusion, by potentially decreasing the proliferation and the migration of the cells isolated from cervical trachea and lung tissues (Ma et al., 2024), CUR-NPs could target two major pathophysiological mechanisms involved in ASM remodeling and could represent a new promising treatment in asthma. Further studies on this issue, including studies in chronic mouse model studies to allow reversal of established remodeling to be assessed, obviously remain necessary and that, published by Ma et al. (2024), is thus an important contribution to identify potential new treatment of airway remodeling.
